# Genetic Characteristics and Variation Spectrum of USH2A-Related Retinitis Pigmentosa and Usher Syndrome

**DOI:** 10.3389/fgene.2022.900548

**Published:** 2022-08-30

**Authors:** Wei Li, Xiao-Sen Jiang, Dong-Ming Han, Jia-Yu Gao, Zheng-Tao Yang, Li Jiang, Qian Zhang, Sheng-Hai Zhang, Ya Gao, Ji-Hong Wu, Jian-Kang Li

**Affiliations:** ^1^ College of Life Sciences, University of Chinese Academy of Sciences, Beijing, China; ^2^ BGI-Shenzhen, Shenzhen, China; ^3^ Department of Ophthalmology, Laizhou City People’s Hospital, Yantai, China; ^4^ Eye Institute, Eye and ENT Hospital, College of Medicine, Fudan University, Shanghai, China; ^5^ Shanghai Key Laboratory of Visual Impairment and Restoration, Science and Technology Commission of Shanghai Municipality, Shanghai, China

**Keywords:** USH2A gene, rp, USH, founder mutations, genetic profile

## Abstract

**Purposes:** We aimed to characterize the USH2A genotypic spectrum in a Chinese cohort and provide a detailed genetic profile for Chinese patients with USH2A-IRD.

**Methods:** We designed a retrospective study wherein a total of 1,334 patients diagnosed with IRD were included as a study cohort, namely 1,278 RP and 56 USH patients, as well as other types of IEDs patients and healthy family members as a control cohort. The genotype-phenotype correlation of all participants with USH2A variant was evaluated.

**Results:** Etiological mutations in USH2A, the most common cause of RP and USH, were found in 16.34% (n = 218) genetically solved IRD patients, with prevalences of 14.87% (190/1,278) and 50% (28/56). After bioinformatics and QC processing, 768 distinct USH2A variants were detected in all participants, including 136 disease-causing mutations present in 665 alleles, distributed in 5.81% of all participants. Of these 136 mutations, 43 were novel, nine were founder mutations, and two hot spot mutations with allele count ≥10. Furthermore, 38.5% (84/218) of genetically solved USH2A-IRD patients were caused by at least one of both c.2802T>G and c.8559–2 A>G mutations, and 36.9% and 69.6% of the alleles in the RP and USH groups were truncating, respectively.

**Conclusion:** USH2A-related East Asian-specific founder and hot spot mutations were the major causes for Chinese RP and USH patients. Our study systematically delineated the genotype spectrum of USH2A-IRD, enabled accurate genetic diagnosis, and provided East Asian and other ethnicities with baseline data of a Chinese origin, which would better serve genetic counseling and therapeutic targets selection.

## Introduction

Usherin (USH2A) variants are associated with retinitis pigmentosa (RP) or usher syndrome (USH) phenotypes. A considerable number of autosomal recessive retinitis pigmentosa (arRP; MIM#268000) or Usher syndrome type II (USH2, MIM#276901) patients are due to USH2A gene defects, and there is an incomplete understanding of the USH2A-related inherited retinal disease (USH2A-IRD) genotype spectrum, which reduces the use of future gene therapy and optimal genetic counseling. According to RetNet (https://sph.uth.edu/retnet/sum-dis.htm), variants in 66 and 16 genes have been associated with arRP and atypical forms of USH, respectively (Date of Visit: December 2021) (Stephen, Daiger, Lori, Sullivan, Sara, Bowne; Reurink et al., 2021). Based on severity and progression, three clinical forms of USH can be distinguished: USH type I (USH1), type II (USH2), and type III (USH3). Of these, USH type II (USH2) is the most common clinical form (Millán et al., 2011).

USH2A deficiencies are estimated to be responsible for 8–19% of arRP patients (Stone et al., 2017; Perea-Romero et al., 2021), 29–55% of all USH patients, and 57–90% of USH type 2 (USH2) patients (McGee et al., 2010; Jouret et al., 2019; Toualbi et al., 2020). The prevalence of arRP and USH is expected to range from 22–67 per 100,000 individuals (Boughman et al., 1980; Puech et al., 1991) and 4–6 per 100,000 individuals (Spandau and Rohrschneider, 2002; Rosenberg et al., 2008), respectively. Especially considering the huge Chinese population, it highlights the importance of screening for USH2A genes in medical diagnostic tests (Hanany et al., 2020). With the extension of the mutation spectrum of USH2A-IRD patients, the theoretical foundation has also been laid, paving the way for the formulation of treatment strategies.

The USH2A gene (OMIM #608400), located on chromosome 1q41, spans 800,503 nucleotides (nt) and contains 73 exons (NM_206,933.2), including cochlear-specific exon 71, which brings challenges to the identification of disease-causing mutations (Van Wijk et al., 2004; Adato et al., 2005). The USH2A gene encodes usherin, a 5,202 amino acid transmembrane protein, which is mainly produced in the photosensitive layer of the retina, the hair cells of the cochlea, and the basement membrane of many tissues (Liu et al., 2007). Different mutations in the USH2A gene are related to a large group of heterogeneous diseases. Apart from the typical USH2, different mutations in USH2A are related to non-syndromic autosomal recessive RP (Lenassi et al., 2015; Ng et al., 2019). Most of the variants in USH2A are rare, and 1,970 distinct public DNA variants have been reported so far, which are distributed throughout the gene (USHbases: http://www.lovd.nl/USH2A) (Roux et al., 2011; Baux et al., 2014). Up to now, more than 600 disease-causing mutations have been reported, some of which are from the same ancestor. These include c.2299delG *p*. (Glu767fs) and c.2276G>T 77 *p*. (Cys759Phe) mutations, both of which reside in exon 13 of the USH2A gene, are derived from 78, a common ancestor, and are therefore seen more frequently in Southern European populations (Pennings et al., 2004; Aller et al., 2010).

The USH2A gene has a large number of protein-encoding amino acids, and many variants of uncertain significance (VUS) will be detected, which makes it difficult to determine the true causal variation. Although many arRP and USH2 patients have been genetically diagnosed with known disease-causing variants, there are still a large number of patients who have not received an informative genetic diagnosis. In addition, the spectrum of USH2A-IRD mutations between different races vary greatly. c.2299delG and c.2276G>T are significantly enriched in southern European populations, and both reside in exon 13 of the USH2A gene, representing 27.8% and 7.1% of all pathogenic USH2A alleles, respectively (Bernal et al., 2003; Blanco-Kelly et al., 2015; Sun et al., 2018; Dulla et al., 2021). The founder mutation c.8559–2 A>G was only detected in Chinese and Japanese patients, accounting for 19.1% of the identified USH2A alleles (Nakanishi et al., 2011; Chen et al., 2014; Jiang et al., 2015). Genetic diagnosis is essential for determining individual clinical symptoms and guiding prognosis, and it is also a prerequisite for determining compliance with any gene-specific therapy. Currently, gene therapy for USH2A-IRD is moving towards a promising future (Fuster-García et al., 2017; Samanta et al., 2019; Sanjurjo-Soriano et al., 2019). A total of three clinical trials (ClinGov ID: NCT03780257/NCT03146078/NCT04820244) related to USH2A are underway, including a phase 1/2 clinical trial (ClinGov ID: NCT03780257, https://clinicaltrials.gov/ct2/show/NCT03780257, Last Update Posted: 15 September 2021). The purpose of this study is to evaluate the safety and tolerability of QR-421 administered in subjects with RP due to mutations in exon 13 of the USH2A gene (Dulla et al., 2021).

To date, a large number of studies have focused on the clinical and genetic characteristics of patients with USH2A mutations. However, most studies were conducted in non-East Asian cohorts (Blanco-Kelly et al., 2015; Lenassi et al., 2015; Reurink et al., 2021), with limited data on Chinese patients, and a lack of knowledge of mutation spectrum differences between other populations (Nakanishi et al., 2011; Ng et al., 2019). In this study, we obtained USH2A variants data of all 8,710 participants. A total of 1,334 IRD patients were included as study cohort, as well as previous studies by our team, with other types of inherited eye diseases (IEDs) patients and healthy family members (*n* = 7,376) as control cohort (Gao et al., 2019; Gao et al., 2021; Li et al., 2021). The aim of this study was to provide detailed genetic characteristics and evidence of East Asian-specific genetic features in USH2A-IRD and provide a genotype spectrum and novel variant classification evidence for Chinese patients with USH2A variants.

## Methods

### Enrolment, Ethical, and Clinical Examination

This large cohort of IRD patients, which was part of the collection from 2016 to 2019 at the Eye and ENT Hospital of Fudan University, included 1,278 RP patients and 56 USH patients (Gao et al., 2019; Gao et al., 2021). USH is a rare genetic disorder that affects hearing and vision. It can cause deafness or hearing loss and an eye disease called RP. The study cohort included 1,334 IRD patients, and the control cohort include 7,376 non-IRD phenotype participants. In these 1,334 IRD patients, which included 350 RP and 23 USH proband-parent trio, the majority of the remaining patients had only either parents or siblings. The Institutional Review Board (IRB) of the Ethics Committee of the Eye, Ear, Nose, and Throat (ENT) Hospital of Fudan University approved the protocol for this study. All participating members provided written informed consent in accordance with the tenets of the Declaration of Helsinki. The informed consent contains the choice to accept or refuse to continue to use the genetic or clinical data for further studies.

A detailed family history of the participant was collected and a complete clinical ocular examination was performed. Complete ocular examination included: slit-lamp biomicroscopic, best-corrected visual acuity (BCVA), color vision examination (Ishihara color plate test), fundus autofluorescence (Spectralis HRA COCT; Heidelberg, Germany), full-field electroretinography (ERG), intraocular pressure (IOP, Goldmann tonometry), Humphrey perimetry (Carl Zeiss Meditec, Inc, Dublin, CA, United States), and spectral-domain optical coherence tomography (SD-OCT) images recorded.

### NGS-Panel and Variant Detection

5 ml peripheral blood were collected from all participating members, and genomic DNA was extracted using the Genomic DNA Extraction Kit (Qiagen, Venlo, The Netherlands). A customized targeted enrichment capture panel designed by Beijing Genomics Institute (BGI-Shenzhen, China) was used to process DNA samples. Genetic testing was performed by targeted exome sequencing as previously reported (Gao et al., 2019; Gao et al., 2021; Li et al., 2021).

Genetic data for the USH2A gene were obtained for all 8,710 participants, and all USH2A variants were sorted and counted. In the study cohort, the focus was on the detection rate of etiological mutations and mutational spectrum of the USH2A gene in 1,334 IRD patients. Patients with IRD caused by two or more pathogenic or likely pathogenic mutations in USH2A were defined as genetically resolved USH2A-IRD. In the remaining control cohort, USH2A variants in the carriers were sorted out, and genotype profiles and allele frequencies were statistically analyzed.

### Filtering and Variant Classification

Sequence variants were called and annotation was performed, and the validity of all USH2A variant nomenclature was verified by studies (gene name, functional change, database frequency, amino acid change, etc.) (Wang et al., 2010). All initial variants were further filtered to obtain high-quality variants. Sequence variants quality control (QC) was performed using the following parameters: 1) population allele frequency filtering, including 1,000 Genomes Project, dbSNP, ESP6500, ExAC, and gnomAD, variants were included with minor allele frequency (MAF) ≤ 0.01; 2) exclude variants if the sequence depth is less than 30X; and 3) exclude variants located in UTR region (Consugar et al., 2015; Li et al., 2021). After obtaining all the high-quality variants, the number of variants types were calculated and counts were made of each alternate allele for each locus across all participants (AC, allele count), and the number of heterozygous and homozygous individuals (Hanany et al., 2020). In this study, nucleotide sites with significantly high mutation frequencies were defined as “hot spot mutations”. Hotspot mutations often reflect specific mechanisms that generate mutations at specific loci, as well as unusual properties of phenotypic selection schemes. Founder mutations are defined as genetic alterations observed at high frequency in geographically or culturally isolated populations in which one or more ancestors are carriers of the altered gene (Zlotogora, 1994; Horton et al., 2021).

The previously reported USH2A variants are determined according to ClinVar, HGMD, and literature reported, otherwise the variants are classified as novel variants. Deleterious effect prediction of the variants used multiple algorithms, including Sorting Intolerant From Tolerant (SIFT), Likelihood Ratio Test (LRT), Mutation Taster, and FATHMM. All variants with a MAF ≤0.01 were evaluated and classified as pathogenic (P), likely pathogenic (LP), variants of uncertain significance (VUS), likely benign (LB), and benign (B) according to the criteria and guidelines of the American College of Medical Genetics and genomics (ACMG) (Richards et al., 2015). Identification of etiological mutations were combined with variants quality, database allele frequency, potential deleterious effects, and mutations reported in ClinVar, HGMD, or literature. The genotype-phenotype correlation analysis was performed according to the Online Mendelian Inheritance in Man (OMIM) database (Stenson et al., 2012; Johnston and Biesecker, 2013). Finally, Sanger sequencing and family co-segregation analysis were performed within family members to verify the pathogenicity of candidate variants.

## Results

### Characteristics of the Participants

**FIGURE 1:**
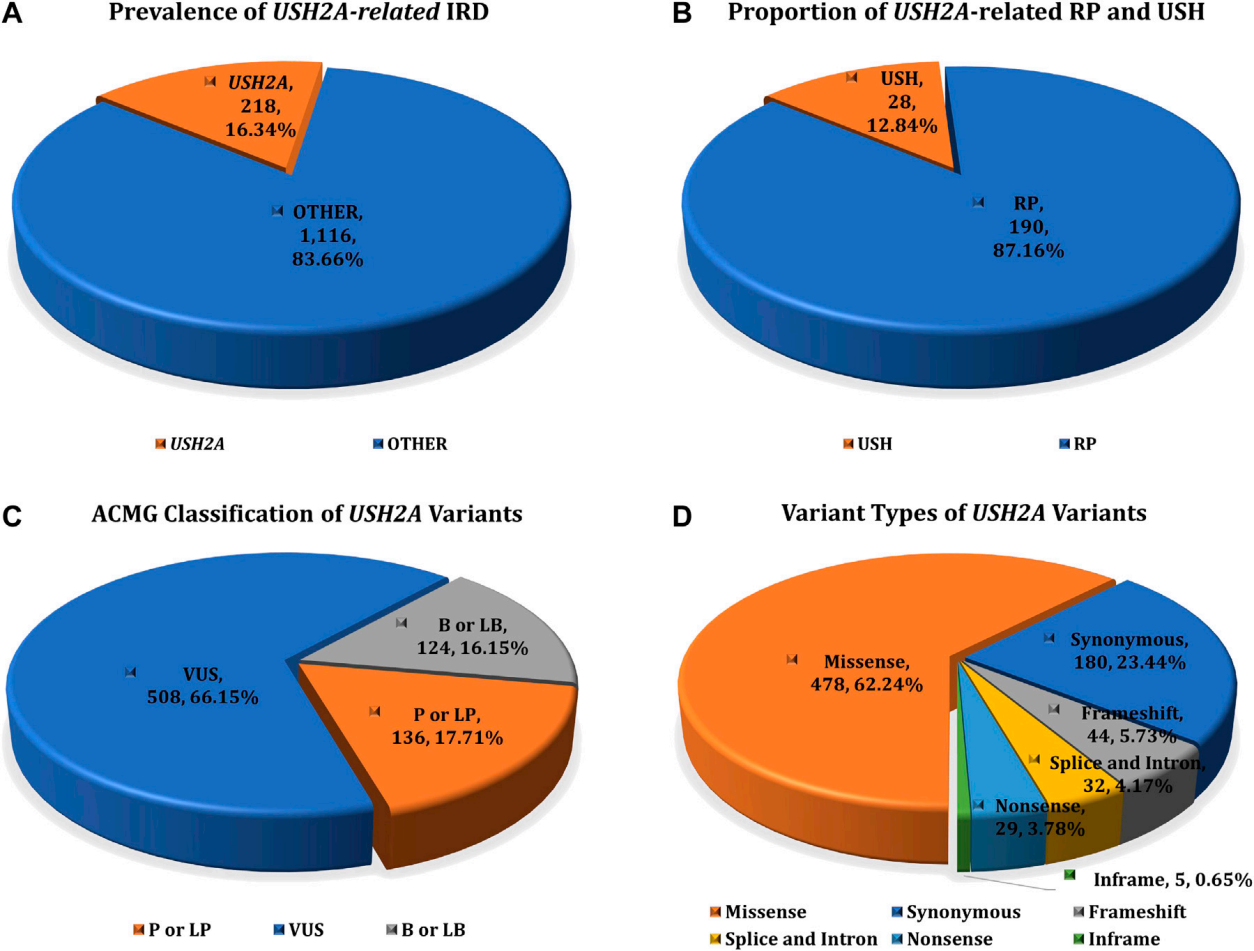
The prevalence of USH2A-IRD and variant category of 768 distinct USH2A variants. **(A)** Homozygous or compound heterozygous pathogenic mutations were detected in 218 (16.34%, 218/1,334) patients with USH2A-IRD; **(B)** 218 genetically solved USH2A-IRD patients, including 190 RP and 28 USH patients; **(C)** ACMG classification of 768 distinct USH2A variants; **(D)** Proportion of six types of 768 distinct USH2A sequence variants. P or LP, pathogenic or likely pathogenic; VUS, variants of uncertain significance; B or LB, benign or likely benign.

The study cohort included 1,334 IRD patients, while the control cohort include 7,376 non-IRD phenotype participants (other IEDs patients and healthy family members), all of whom were screened for USH2A gene variants. After variant pathogenicity interpretation and genotype-phenotype correlation analysis, 218 IRD patients were detected with two or more pathogenic or likely pathogenic mutations in the USH2A gene, accounting for 16.34% (218/1,334) of all IRD patients. These IRD patients were defined as genetically solved USH2A-IRD. The prevalence of RP and USH patients with USH2A homozygous or compound heterozygous mutations was 14.87% (190/1,278) and 50% (28/56), making USH2A the most frequently mutated gene in study cohort (Figure 1A,B).

A total of 5,636 USH2A alleles were identified in all IRD and non-IRD phenotype participants. These variants are classified as P or LP, VUS, B or LB, and allele frequencies in the 1,000 Genomes Project, dbSNP, ESP6500, ExAC, and gnomAD databases with minor allele frequency (MAF) ≤ 0.01. Variant pathogenicity was analyzed by genotype-phenotype information, including clinical diagnosis, familial inheritance pattern, and ACMG guidelines.

### USH2A-Related Variants Spectrum

USH2A sequencing was performed by targeted exome sequence in all 8,710 participants, identifying 768 distinct sequence variants, present in 5,636 alleles in all participants. Among these 768 variants, we found 318 variants with ClinVar annotations, and 209 variants with Leiden Open Variation Database (LOVD) annotations. In total, 768 USH2A variants were classified according to the ACMG guidelines, including 136 P or LP variants, 508 VUS variants, and 124 B or LB variants (Figure 1C). These 768 distinct variants included 478 missense (AC = 3,627), 44 frameshift (AC = 114), 29 nonsense (AC = 60), 32 intron and splice (AC = 644), five inframe (AC = 7), and 180 synonymous (AC = 1,184) variants (Figure 1D). The complete details of these variants are given in Supplementary Table S1.

**FIGURE 2:**
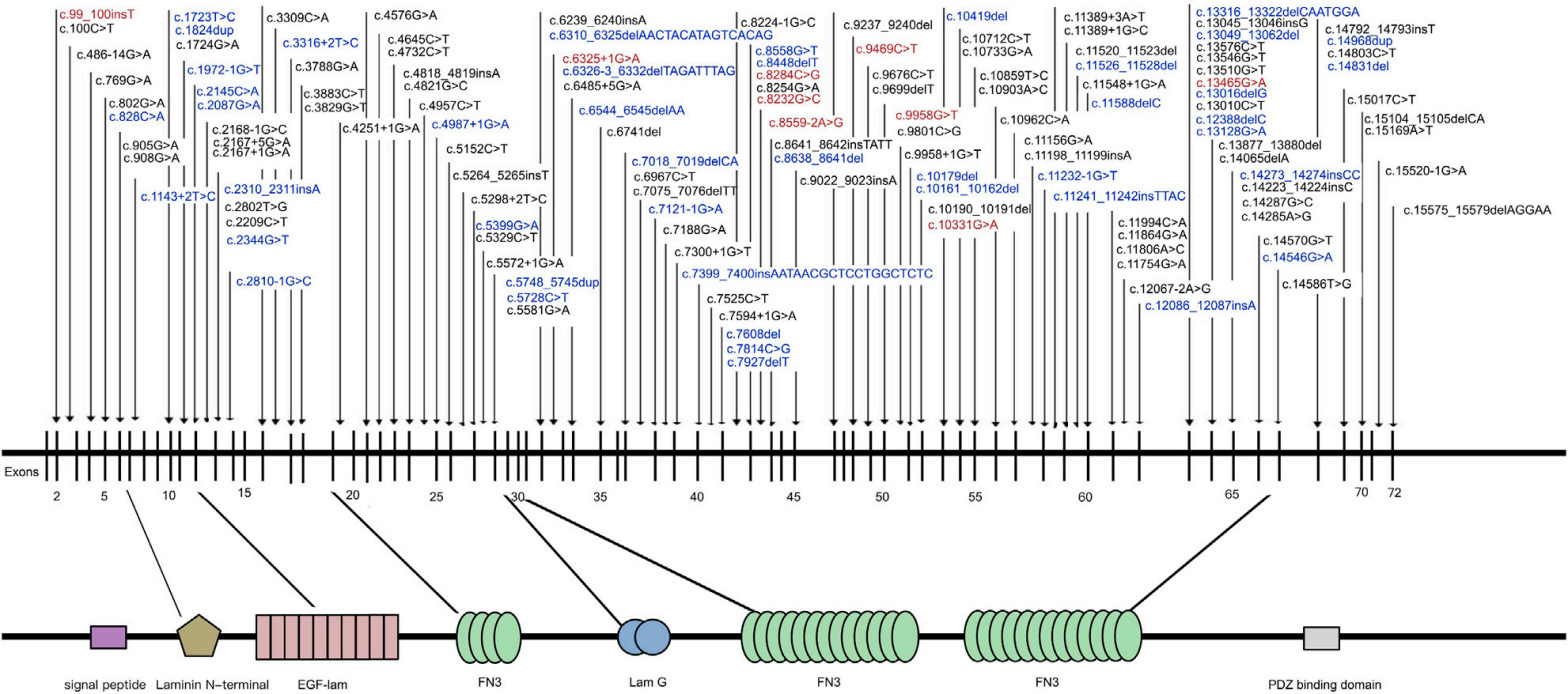
Naturally occurring mutations of USH2A detected in patients with IRD. Blue indicates novel mutations first reported in this study; Red indicates mutations were founder mutations identified in East Asian Populations.

In the study cohort, 218 genetically solved patients with USH2A-IRD were identified, with a total of 444 W disease-associated alleles detected. Of these 218 genetically solved patients, 23 (10.55%) were homozygous and 195 (89.45%) were compound heterozygous mutations. The most frequent mutations were c.2802T>G and c.8559–2 A>G, detected in 50 and 47 USH2A-IRD patients, respectively. Overall, 38.5% (84/218) of USH2A-IRD patients had at least one of these two most frequent mutations. Details of the 444 disease-associated alleles identified in 218 USH2A-IRD patients are given in Supplementary Table S2. According to the ACMG criteria, 136 variants were considered as P or LP mutations, of which 43 were novel, as along with nine founder mutations and two hotspots mutations (AC> = 10). All identified mutations spread in the entire gene and impacted all functional domains (Figure 2). Taken together, the sequence variant spectrum includes 35 missense variants, two of which may also affect splicing, 29 nonsense, 27 intron-exon splicing, 44 frameshift, and one inframe_deletion variant (Supplementary Table S3). Of these P or LP mutations, 73.5% (100/136) were truncating mutations, and the remainder were missense mutations with one inframe_deletion mutation.

In particular, we detected three variants previously reported in the LOVD or ClinVar databases. The first variant, c.15520-1G >A, was detected in patient P131, annotated as “Conflicting interpretations of pathogenicity” in ClinVar, but absent in LOVD. A second variant, c.10859T>C/*p*.Ile3620Thr, was detected in patients P13, P108, and P196, defined as “Conflicting interpretations of pathogenicity” in ClinVar and “P, LP, VUS” in LOVD. This mutation has a population frequency of 1.16E-04 in the gnomAD-EAS database and was predicted to be deleterious in the SIFT, LRT, and MutTaster algorithms. The c.10859T>C/*p*.Ile3620Thr mutation was reported as the disease-causing mutation in multiple RP families and verified by co-segregation. In two Japanese RP patients, compound heterozygous mutations c.10859T>C/*p*.Ile3620Thr; c.14766G>A/*p*.Trp4922* and c.10859T>C/*p*.Ile3620Thr; c.11328T>G/*p* .Tyr3776* were detected, respectively (Inaba et al., 2020). In a Chinese family RP-F6, mutations c.10859T >C/*p*.Ile3620Thr and c.12880delA/*p*.I4294Lfs*21 were detected in patients II -3, which were inherited from parents, respectively (Qu et al., 2020). In a Chinese study of RP and USH caused by USH2A mutation, compound heterozygous mutation c.10859T>C/*p*.Ile3620Thr; c.8559–2 A G/- was detected in one USH patient. Otherwise, compound heterozygous mutations c.10859T>C/*p*.Ile3620Thr; c.8559–2 A>G/-, and c.7569G>A/*p*.Trp2523* were detected in another two RP patients. In this study, c.10859T>C/*p*.Ile3620Thr was defined as the LP mutation (Meng et al., 2021). A third variant, c.4251 + 1G>A, annotated as “VUS” in the LOVD and ClinVar database, was detected in one patient P32. However, our expert panel evaluated and reclassified all three variants as P or LP mutations, according to the aforementioned criteria and guidelines of the ACMG (Table 1). The complete details of these three variants are provided in Supplementary Tables S1, S2.

**TABLE 1:** Three variants were reclassified as P or LP mutations according to ACMG guidelines in our USH2A-IRD study cohort.

Patients ID	Mutant 1	Mutant 2	Mutant 1 in gnomAD-EAS	Mutant 1 in SIFT, LRT, muttaster, FATHMM	Mutant 1 in clinvar	Mutant 1 in LOVD	ACMG Re-classified
P131	c.15520-1G>A, -	c.10331G>A, *p*.Cys3444Tyr	NA	-,-,D,-	Conflicting interpretations of pathogenicity	Novel	LP
P13	c.10859T>C, *p*.Ile3620Thr	c.13465G>A, *p*.Gly4489Ser	1.16E-04	D,D,D,T	Conflicting interpretations of pathogenicity	P, LP, VUS	LP (Inaba et al., 2020; Qu et al., 2020; Meng et al., 2021)
P108		c.10712C>T, *p*.Thr3571Met	1.16E-04	D,D,D,T			
P196		c.6071C>T, *p*.Pro2024Leu	1.16E-04	D,D,D,T			
P32	c.4251 + 1G>A, -	c.3316 + 2T>C, -	NA	-,-,D,-	VUS	VUS	P

P or LP, pathogenic or likely pathogenic; VUS, variants of uncertain significance.

### USH2A-Related Founder and hot Spot Mutations

Of these 136 mutations, 78 were absent in ClinVar (consulted in December 2021) or 70 were absent in LOVD (consulted in December 2021). In addition, we assessed the AC of 136 disease-causing variants to summarize the variants frequently found in this Chinese cohort. Eleven frequent mutations were detected with an AC≥10, and these top eleven mutations accounted for 57.74% (384/665) of total alleles (Table 2, Supplementary Table S3). The most frequent mutationswas c.2802T>G/*p*.Cys934Trp (AC = 122), which was detected in 22.94% (50/218) USH2A-IRD patients. We note that 130 of 218 (59.63%) USH2A-IRD patients carried at least one of these eleven frequent mutations (Supplementary Table S2).

**TABLE 2:** Top eleven frequent mutations with an allele count ≥10.

Chr:Por:mut	Mutant name	Amino acid change	Our 8,710 cohort			East asian ALT freq			Non-east asian ALT freq	Reported ethnic group	Founder or hot spot mutations?
			Het-hom (n0	AC(n)	ALT Freq (AC/17,420)	G1000_EAS	gnomAD_EAS (gnomAD V3.1.1)	ExAC_EAS			
chr1:216246592:A>C	c.2802T>G	*p*.Cys934Trp	120–1	122	0.0070	0.004	3.27E-03	2.79E-03	8.66E-05 (ExAC_AMR) 1.50E-05 (ExAC-NFE) 2.89E-04 (gnomAD_ASJ)	China (Gao et al., 2021); Britain (Lenassi et al., 2015); Japan (Inaba et al., 2020)	Hot Spot Mutation
chr1:215877882:T>C	c.8559–2 A>G	_	83–8	99	0.0057	0.001	1.93E-04	3.48E-04	-	China (Chen et al., 2014; Sun et al., 2020; Gao et al., 2021); Japan (Nakanishi et al., 2011)	Founder Mutation (27–28)
chr1:216422237:G>GA	c.99_100insT	*p*.Arg34Serfs41	23–2	27	0.0015	0	3.86E-04	0	-	China (Gao et al., 2021); Britain (Lenassi et al., 2015)	Founder Mutation
chr1:215759735:C>T	c.11156G>A	*p*.Arg3719His	23–2	27	0.0015	0	1.93E-04	1.16E-04	1.69E-04 (gnomAD_AFR) 1.47E-05 (gnomAD_NFE) 2.60E-04 (ExAC_AMR) 1.92E-04 (ExAC_AFR) 6.06E-05 (ExAC_SAS)	China (Gao et al., 2021); Britain (Lenassi et al., 2015); America (McGee et al., 2010)	Hot Spot Mutation
chr1:215879090:C>G	c.8232G>C	*p*.Trp2744Cys	16–2	20	0.0011	0	1.93E-04	0	-	China (Sun et al., 2020; Gao et al., 2021)	Founder Mutation
chr1:215674446:C>T	c.13465G>A	*p*.Gly4489Ser	16–1	18	0.0010	0	0	0	-	China (Gao et al., 2021)	Founder Mutation
chr1:215879038:G>C	c.8284C>G	*p*.Pro2762Ala	12–2	16	0.0009	0	0	0	-	China (Chen et al., 2014; Gao et al., 2021)	Founder Mutation
chr1:215798907:C>A	c.9958G>T	*p*.Gly3320Cys	12–2	16	0.0009	0	0	0	-	China (Chen et al., 2014)	Founder Mutation
chr1:215786726:C>T	c.10331G>A	*p*.Cys3444Tyr	14–0	14	0.0008	0	5.44E-05	0	-	China (Gao et al., 2021)	Founder Mutation
chr1:215817098:G>A	c.9469C>T	*p*.Gln3157*	14–0	14	0.0008	0	1.94E-04	3.48E-04	-	China (Sun et al., 2020; Gao et al., 2021), Japan (Nakanishi et al., 2011)	Founder Mutation
chr1:216046430:C>T	c.6325 + 1G>A	_	11–0	11	0.0006	0	0	0	-	China (Sun et al., 2020)	Founder Mutation

AC, allele count; ALT, alternative allele; Freq, Frequency; Het-Hom, number of participants with heterozygous or homozygous forms; Chr:por:mut, Chromosome_Position_Mutant; G1000_EAS_AF, 1000 Genomes Project East Asian Allele Frequency; gnomAD_EAS_AF, genome aggregation database east asian allele frequency; ExAC_EAS_AF, exome aggregation consortium east asian allele frequency; AMR, American Admixed/Latino; NFE, Non-Finnish European; ASJ, ashkenazi jewish; AFR, African/African American; SAS, South Asian.

The founder mutation c.8559–2 A>G was detected in 91 participants (AC = 99), made up of eight homozygous mutation USH2A-IRD patients and 83 heterozygous mutation carriers. In short, at least one c.8559–2 A>G mutant allele was detected in 21.56% (47/218) USH2A-IRD patients. According to the G1000, ExAC, and gnomAD database, this mutation was not found in non-East Asian populations, and was specifically enriched in East Asian populations (G1000_EAS = 0.001, gnomAD_EAS = 0.000193, ExAC_EAS = 0.000348) (Table 2). The most frequent mutation, c.2802T>G, was detected in 121 participants, and only one participant was of a homozygous form. c.2802T>G mutation is highly enriched in East Asian populations, with an alternative allele frequency of 0.004 in 1000 Genomes Project East Asian database or 0.00279 in Exome Aggregation Consortium East Asian database. Interestingly, the c.2802T>G mutation was also occasionally found in non-East Asian populations and was found in the Ashkenazi Jewish population in the gnomAD database, and American Admixed/Latino and Non-Finnish European populations in the ExAC database. In sum, based on the above population characteristics and finding, we define it as a hot spot mutation (Table 2) (Gao et al., 2021).

In addition to these two highly frequent mutations, we detected nine other frequent mutations with an allele count ≥10, including one novel hot spot mutation c.11156G>A (*p*.Arg3719His), and eight novel founder mutations (c.99_100insT; c.8232G>C; c.13465G>A; c.8284C>G; c.9958G>T; c.10331G>A; c.9469C>T; c.6325 + 1G>A) (Table 2).

### RP and USH Related USH2A Variants

**FIGURE 3:**
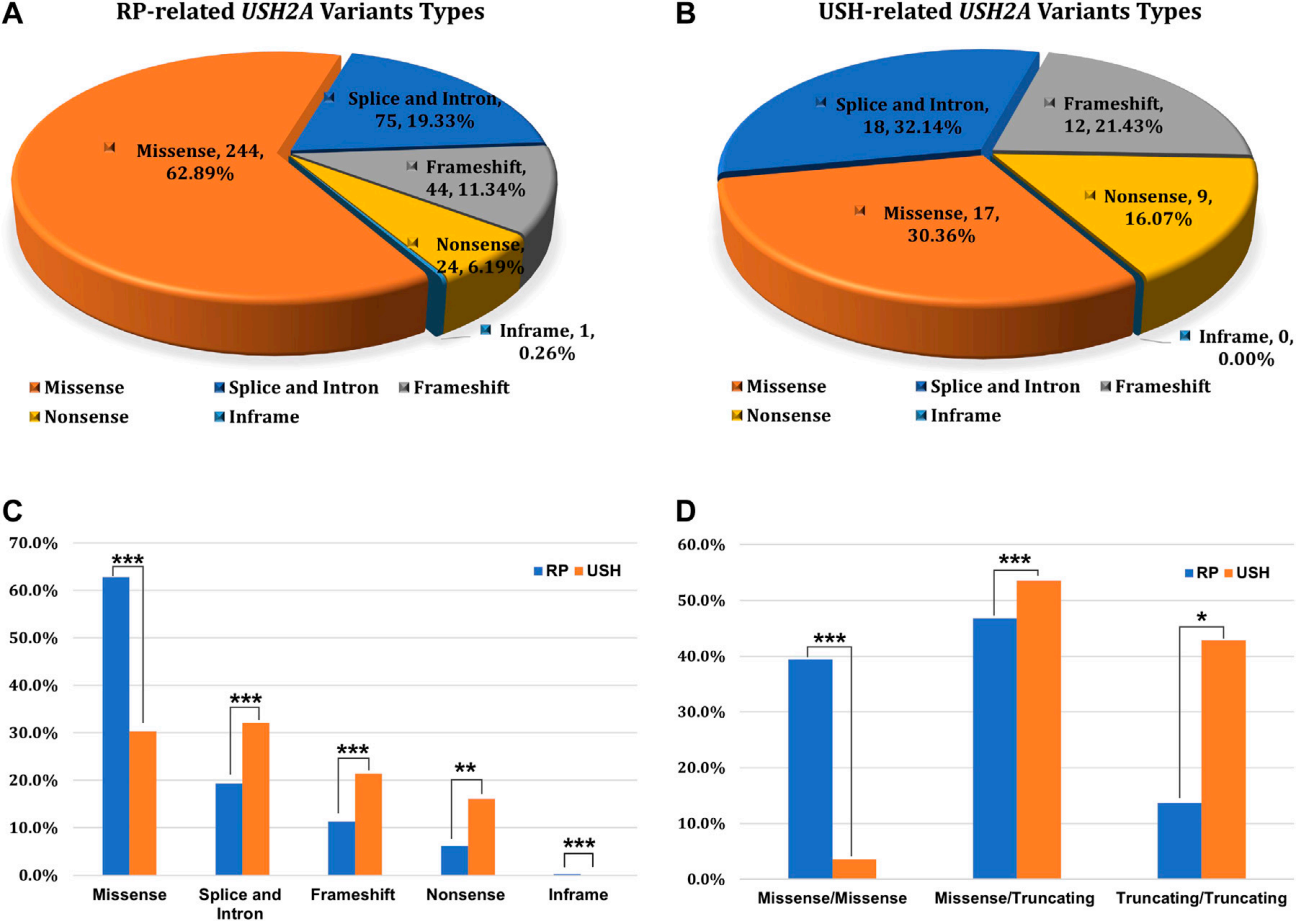
The type of variation and the distribution of genotype subgroups. **(A)** Variation types of 388 alleles in USH2A-related RP patients (*n* = 190); **(B)** Variation types of 56 alleles in USH2A-related USH patients (*n* = 28); **(C)** Allele ratios of distinct variant types between RP and USH, missense group (244 vs. 17), splice and intron group (75 vs. 18), frameshift group (44 vs. 12), nonsense group (24 vs. 9), inframe group (1 vs. 0); **(D)** Comparison of variant combinations between RP and USH patients, missense/missense group (75 vs. 1), missense/truncating group (89 vs. 15), and truncating/truncating group (26 vs. 12). χ^2^test: *p < 0.05; **p < 0.01; ***p < 0.001.

Of the 1,334 patients diagnosed with IRD, 218 patients received a USH2A-related genetic diagnosis which could explain their symptoms. Overall, 444 alleles were detected in 218 genetically solved USH2A-IRD patients, including 388 alleles in 190 patients with RP and 56 alleles in 28 patients with USH. Overall, 230 missense variants (AC = 244, 62.9%), 67 splice and intron variants (AC = 75, 19.3%), 42 frameshift variants (AC = 44, 11.3%), 24 nonsense variants (AC = 24, 6.2%), and one inframe variant (AC = 1, 0.3%) were detected in USH2A-related RP patients (Figure 3A). Seventeen missense variants (AC = 17, 30.4%), 17 splice and intron variants in 18 alleles (AC = 18, 32.1%), 12 frameshift variants (AC = 12, 21.4%), nine nonsense variants (AC = 9, 16.1%), and no inframe variant were detected in USH2A-related USH patients (Figure 3B).

The most frequent mutations, c.2802T>G (AC = 51) and c.8559–2 A>G (AC = 55), were identified in 50 and 47 genetically solved USH2A-IRD patients, respectively. For these two mutations, 9.63% (21/218) of genetically solved IRD patients were simultaneously detected in the form of compound heterozygotes or homozygotes. In addition, we studied the variation spectrum of genetically solved USH2A-IRD patients and assessed whether the identified pathogenic variants were enriched in RP or USH patients. Table 3 presented the top eleven frequent mutations in 218 genetically solved patients with USH2A-IRD. The results showed that ten variants with alternative allele frequency (ALT Freq) > 0.01 were significantly enriched in genetically solved patients with RP, including hot spot mutation c.2802T>G and reported founder mutation c.8559–2 A>G, both of which have an alternative allele frequency greater than 0.01. In the genetically solved patients with USH, six variants were significantly enriched with allele frequency >0.01, and five variants were not detected. In particular, the enrichment of the four variants (c.8559–2 A>G, c.8232G>C, c.9469C>T, and c.6325 + 1G>A) in USH patients was significantly greater than that of RP.

**TABLE 3:** Top eleven frequent mutations in 218 genetically solved patients with USH2A-IRD, including 190 patients with RP and 28 patients with USH.

Chr:Position:Allele	Mutant name	Amino acid change	Total 218 genetically solved IRD patients				Solved patients with RP			Solved patients with USH		
			Patients (n)	Het-hom (n)	AC (n)	ALT Freq (AC/436)	Patients (n)	AC (n)	ALT Freq (AC/380)	Patients (n)	AC (n)	ALT Freq (AC/56)
chr1:216246592:A>C	c.2802T>G	*p*.Cys934Trp	50	49–1	51	0.1170	47	48	0.1263	3	3	0.0536
chr1:215877882:T>C	c.8559–2 A>G	_	47	47–8	55	0.1261	40	47	0.1237	7	8	0.1429
chr1:216422237:G>GA	c.99_100insT	*p*.Arg34Serfs41	12	10–2	14	0.0321	11	13	0.0342	1	1	0.0179
chr1:215759735:C>T	c.11156G>A	*p*.Arg3719His	10	10–2	12	0.0275	10	12	0.0316	0	0	0.0000
chr1:215879090:C>G	c.8232G>C	*p*.Trp2744Cys	7	7–2	9	0.0206	5	7	0.0184	2	2	0.0357
chr1:215674446:C>T	c.13465G>A	*p*.Gly4489Ser	9	9–1	10	0.0229	9	10	0.0263	0	0	0.0000
chr1:215879038:G>C	c.8284C>G	*p*.Pro2762Ala	6	6–2	8	0.0183	6	8	0.0211	0	0	0.0000
chr1:215798907:C>A	c.9958G>T	*p*.Gly3320Cys	6	6–2	8	0.0183	6	8	0.0211	0	0	0.0000
chr1:215786726:C>T	c.10331G>A	*p*.Cys3444Tyr	3	3–0	3	0.0069	3	3	0.0079	0	0	0.0000
chr1:215817098:G>A	c.9469C>T	*p*.Gln3157*	7	7–0	7	0.0161	4	4	0.0105	3	3	0.0536
chr1:216046430:C>T	c.6325 + 1G>A	_	7	7–0	7	0.0161	4	4	0.0105	3	3	0.0536

AC, allele count; ALT, alternative allele; Freq, Frequency; Het-Hom, number of participants with heterozygous or homozygous forms; Chr:por:mut, Chromosome_Position_Mutant.

Notably, three types of variant combination forms in 218 USH2A-IRD patients were statistically analyzed, including 77 (35.32%) missense/missense, 112 (51.38%) missense/truncating, and 29 (13.30%) truncating/truncating patients (Figure 3C,D). The mean age was 41.13 ± 16.12 years (range, 2–78 years; median, 41 years) in 218 genetically solved USH2A-IRD patients. Interestingly, compared with detecting only missense mutations, patients with at least one truncating mutation (nonsense, frameshift, splicing) had a younger age at the initial clinical visit. For example, the missense/missense group is 43.73 ± 17.00 years (range, 4–78 years; median, 47 years), the missense/truncating group is 40.75 ± 16.18 years (range, 2–72 years; median, 42 years), and the truncating/truncating group is 36.04 ± 12.30 (range, 10–56 years; median, 35 years). In addition, the types of variant combinations of RP and USH patients are significantly different. It is observed that only 3.6% (1/28) of USH2A-related USH patients had a variant combination of missense/missense pattern, while 40% (76/190) of USH2A-related RP patients are missense/missense.

### *De novo* Variants Analysis

Overall, 373 IRD trios were recruited, including 350 RP and 23 USH proband-parent trios for USH2A gene *de novo* analysis. It is extremely rare that only one *de novo* mutation was detected. 45-year-old women was diagnosed with binocular RP at her first visit. Her 78-year-old father and 74-year-old mother had a normal phenotype (P75). Targeted sequencing was performed on the proband and parents, and compound heterozygous mutations (c.2802T>G and c.8559_2 A>G) of the USH2A gene were detected in the proband. Through trio co-segregation analysis, we found that the mutation c.8559_2 A>G was inherited from the mother, and the mutation c.2802T>G was a *de novo* mutation.

## Conclusion

In this study, we performed targeted exome sequencing on 8,710 participants, of which 1,334 patients were initially diagnosed as RP or USH. A definite molecular diagnosis was obtained in 16.34% (218/1,334) of USH2A-IRD patients, and the prevalence in RP and USH were 14.87% (190/1,278) and 50% (28/56), respectively. Despite ethnic differences in causal genes, USH2A-IRD is still one of the most prevalent IRDs in Caucasian, Japanese, and other populations (Koyanagi et al., 2019; Pontikos et al., 2020). According to our previous report, this proportion is 15.75% (163/1,381) in the Chinese population, which was ranked as the most frequent mutation in Chinese IRD patients (Gao et al., 2019; Gao et al., 2021). The detection rate of this study is higher than previously reported, mainly attributed to the difference in samples recruitment, and the large sample verification has effectively increased the detection rate. USH2A is the second major causative gene in the Japanese population, accounting for 3.82% (46/1,204) of RP patients (Koyanagi et al., 2019). Studies have also reported that pathogenic mutations of USH2A were identified in 6.9% (36/525) of Japanese IRD patients (Inaba et al., 2020).

In total, 768 distinct USH2A sequence variants were identified, including 136 disease-causing mutations that were detected in 665 alleles, distributed among 5.81% (506/8,710) participants. This means that 5.81% of participants have at least one disease-causing mutation, indicating that the carrying rate is 581/10,000 in Chinese IEDs population. We also found that East Asian-specific founder mutations in USH2A genes were the major causes of RP or USH in the Chinese population. The toop eleven frequent mutations with an AC≥10 were identified in our 8,710 IEDs cohort (Table 2). Interestingly, most of these mutations are East Asian-specific, with high East Asian alternative allele frequencies, including one reported and eight novel founder mutations, and two hot spot mutations.

To date, more than 600 disease-causing mutations have been reported, and research on the spectrum of USH2A-related mutations in the Chinese ethnicity has grown significantly. In 11 Chinese RP and USH families, 21 P or LP mutations were identified, including 13 novel mutations (5 with RP and 8 with USH) 46. Among 284 patients (131 diagnosed with USH and 153 diagnosed with RP) with USH2A disease-causing variants in 260 Chinese families, a total of 230 distinct P, LP, or VUS variants were identified, of which 90 (39.13%) have not been reported previously (Zhu et al., 2021). In 69 Chinese patients (36 diagnosed with USH and 33 diagnosed with RP) caused by USH2A disease-causing variants, 79 distinct variants were detected, including 23 novel P, LP, or VUS variants (Meng et al., 2021). A total of 8,710 participants were enrolled in our study, made up of the study cohort of 1,334 IRD patients and the control cohort of 7,376 participants with non-IRD phenotypes. Ultimately, 768 distinct USH2A variants were identified, including 136 P or LP variants, of which 43 P or LP variants had not been reported previously. As well as 508 VUS variants, 403 absent in LOVD or 372 absent in ClinVar database, their pathogenicity needs to be further studied (Supplementary Table S1).Of these top eleven frequency alleles, the most frequent mutation is c.2802T >G (AC = 122), and the alternative allele frequencies in our 8,710 IEDs cohort is 0.007. The frequency of alternative alleles is significantly higher than that of the G1000_EAS (0.004) or ExAC_EAS (0.00279) database, mainly because our study recruited samples from the IEDs cohort, and the disease-causing mutation carrier rate is higher. In addition, the c.2802T >G mutation is occasionally detected in non-East Asian populations, including Ashkenazi Jewish, American Admixed/Latino, and Non-Finnish European populations in the ExAC or gnomAD database. Moreover, the c.2802T>G mutation has also been reported in China, British, and Japan ethnic groups, and we speculate that it is likely to be characterized as a hotspot mutation (Lenassi et al., 2015; Inaba et al., 2020; Gao et al., 2021). The c.8559–2 A >G mutation has been widely reported in USH patients in China and Japan, and is highly enriched in the East Asian population database of G1000, ExAC, and gnomAD, but has not been detected in non-East Asian populations (Nakanishi et al., 2011; Chen et al., 2014; Sun et al., 2020; Gao et al., 2021). c.8559–2 A >G mutation was defined as founder mutation, frequently detected in a majority of Chinese and Japanese USH2 patients (Fuster-García et al., 2017; Sanjurjo-Soriano et al., 2019). All in all, nine founder mutations and two hotspot mutations were identified (Table 2).

This study reports eight novel founder and one novel hotspot mutation for the first time, elucidates the genetic structural differences between races, and presents the unique genetic characteristics of Chinese races. The most frequent USH2A-related mutations in 1,204 Japanese RP patients are c.8254G>A (*p*.Gly2752Arg) and c.2802T>G (*p*.Cys934Trp), which are not reported or extremely rare in non-East Asian populations (MAF <0.01%), with one exception, *p*.Cys934Trp, which was 0.05% in Ashkenazi Jewish population of gnomAD. Both of these mutations were significantly enriched in East Asian populations (MAF> 0.1%) and were detected in more than 10 RP patients (Koyanagi et al., 2019). In the mutation spectrum of our Chinese population, c.2802T>G (*p*.Cys934Trp) is the most frequent hotspot mutation, but c.8254G>A (*p*.Gly2752Arg) is not frequent enough, and only two USH2A-IRD patients were detected with this mutation. The USH2A c.2299delG mutation is the most common founder mutation in the Dutch population, but this mutation was not detected in our Chinese cohort (Aller et al., 2010). The top two frequency mutations (c.2802T>G and c.8559–2 A>G) are widely reported in East Asian populations, but we have not yet known the proportion of these two mutations in RP and USH patients who have received molecular diagnosis. Our study demonstrated that 38.5% (84/218) of genetically solved USH2A-IRD patients were caused by at least one of the c.2802T >G and c.8559–2 A>G mutations. C.2802T>G and c.8559–2 A>G mutations have extremely high allele frequencies in our 8,710 participants, with allele carrier rates being 0.0070 and 0.0057, respectively. Among these genetically solved RP and USH patients with USH2A mutations, five mutations (c.2802T>G, c.8559–2 A>G, c.8232G>C, c.9469C>T, and c.6325 + 1G>A) were more enriched in USH patients, while four mutations (c.2802T>G, c.8559–2 A>G, c.99_100insT, and c.11156G>A) were more enriched in RP patients. Our findings highlight that East Asian-specific founder mutations as well as hot spot mutations in the USH2A gene largely affect RP and USH patients in the Chinese population.

Of these USH2A-related RP patients, three patients (P97, P165, and P218) were found to carry three P or LP variants. In P97 patients, the c.8559–2 A>G mutation was inherited from the father, while both c.9958G>T and c.8284C>G were inherited from the mother. In the P165 patient, three mutations were detected, including a frameshift mutation c.11520_11523del, an inframe_deletion mutation c.11526_11528del, and a missense mutation c.9801C>G. However, parental samples were not obtained and co-segregation analysis could not be performed. In P218 patient, two missense mutations, c.8284C>G and c.9958G>T, and one nonsense mutation, c.9676C>T, were detected, and parental samples were not obtained for co-segregation analysis. The c.8284C>G and c.9958G>T mutations were detected in a homozygous form in one family from China, and were also reported in a heterozygous form in other families (Chen et al., 2014; Gao et al., 2019; Gao et al., 2021). The former causes the amino acid to change from proline to alanine, and the latter changes from glycine to cystine. These two mutations tend to occur simultaneously in homozygous or heterozygous forms, and their underlying pathogenic mechanisms remain to be further studied. In Supplementary Table S2, a total of six patients were detected with c.8284C>G and c.9958G>T, and all patients were clinically diagnosed as RP. Besides P97 and P218 mentioned above, the other four patients are P99, P142, P170, and P211. In P142 patient, three missense heterozygous mutations, c.8284C>G, c.9958G>T, and c.4021G> C, were detected. Similarly, c.8284C>G, c.9958G> T was from the mother and c.4021G>C was from the father. In P170 and P211 patients, homozygous mutations c.8284C>G and c.9958G > T were detected, both of which were sporadic patients, and parental samples were not validated. In P99 patient, heterozygous mutations c.8284C>G and c.9958G>T were detected, but parental samples were not obtained for co-segregation verification. Thus, a possibility that these two mutations are on the same allele and that they may not be the cause of the disease remains. Although we included P99 in the statistics of genetically solved IRD patients, its etiology is currently unknown, and further attention to these two mutations is required by other investigators.

Notably, the detection rate of truncating mutations is significantly enriched in USH patients, and at least one truncated mutation was detected in 96.4% USH patients, and the missense/missense mutation combination was detected in only one patient. Overall, truncation mutations accounted for 36.9% (143/388) and 69.6% (39/56) of alleles in RP and USH patients. In a Chinese study of USH2A-related mutation, 17 missense/missense mutation combinations were detected in 117 USH patients, accounting for 14.5% (17/117) (Zhu et al., 2021). In another Japanese truncation mutation study, the missense/missense mutation combination was not detected in 25 USH patients (Inaba et al., 2020). Nevertheless, in this study and the Japan study, truncation mutations were detected in almost all USH patients, emphasizing that truncation mutations can cause more severe phenotypes including hearing.

The benefit of this large cohort is that we were able to assess population-level variability in USH2A-related IRD disease. Information in our cohort, including co-segregation, mutational hotspot regions, expected frequencies of causal USH2A variants, and significant enrichment in disease cohorts, adds significant evidence for variant classification and will also provide additional criteria for variants entries already present in ClinVar database. Overall, this cohort is a robust baseline dataset that allows calculation of USH2A-related disease-specific allele frequencies, and by comparison with publicly available general population frequencies, we were able to calculate statistical enrichment for disease associations for each variant.

To our knowledge, this is the largest genetic spectrum analysis of USH2A-IRD in the Chinese population. Our genetic data present that USH2A-related East Asian-specific founder mutations and hot spot mutations were the major causes for Chinese RP and USH. A total of 768 distinct sequence variants were identified, as well as nine founder mutations and two hot spot mutations with AC ≥ 10. Our findings provide insights into the differences in the genetic background of RP and USH patients and help to better understand the genetic etiology of RP and USH. Our baseline data will serve as a well-founded reference for genetic counseling and better management for USH2A-IRD patients and putative therapeutic approaches.

## Data Availability

The data that support the findings of this study have been deposited in the CNSA (https://db.cngb.org/cnsa/) of CNGBdb with accession code CNP CNP0000503.

## References

[B1] Adato A. Lefèvre G. Delprat B. Michel V. Michalski N. Chardenoux S. (2005). Usherin, the defective protein in Usher syndrome type IIA, is likely to be a component of interstereocilia ankle links in the inner ear sensory cells. Hum. Mol. Genet. 14, 3921–3932. 10.1093/hmg/ddi416 16301217

[B2] Aller E. Larrieu L. Jaijo T. Baux D. Espinos C. Gonzalez-Candelas F. (2010). The USH2A c.2299delG mutation: Dating its common origin in a southern European population. Eur. J. Hum. Genet. 18, 788–793. 10.1038/ejhg.2010.14 20145675 PMC2987359

[B4] Baux D. Blanchet C. Hamel C. Meunier I. Larrieu L. Faugère V. (2014). Enrichment of LOVD-USHbases with 152 USH2A genotypes defines an extensive mutational spectrum and highlights missense hotspots. Hum. Mutat. 35 (10), 1179–1186. 10.1002/humu.22608 24944099

[B5] Bernal S. Ayuso C. Antiñolo G. Gimenez A. Borrego S. Trujillo M. J. (2003). Mutations in USH2A in Spanish patients with autosomal recessive retinitis pigmentosa: High prevalence and phenotypic variation. J. Med. Genet. 40 (1), e8. 10.1136/jmg.40.1.e8 12525556 PMC1735247

[B6] Blanco-Kelly F. Jaijo T. Aller E. Avila-Fernandez A. López-Molina M. I. Giménez A. (2015). Clinical aspects of Usher syndrome and the USH2A gene in a cohort of 433 patients. JAMA Ophthalmol. 133 (2), 157–164. 10.1001/jamaophthalmol.2014.4498 25375654

[B7] Boughman J. A. Conneally P. M. Nance W. E. (1980). Population genetic studies of retinitis pigmentosa. Am. J. Hum. Genet. 32, 223–235. 7386458 PMC1686021

[B8] Chen X. Sheng X. Liu X. Li H. Liu Y. Rong W. (2014). Targeted next-generation sequencing reveals novel USH2A mutations associated with diverse disease phenotypes: Implications for clinical and molecular diagnosis. Plos One 9 (8), e105439. 10.1371/journal.pone.0105439 25133613 PMC4136877

[B9] Consugar M. B. Navarro-Gomez D. Place E. M. Bujakowska K. M. Sousa M. E. Fonseca-Kelly Z. D. (2015). Panel-based genetic diagnostic testing for inherited eye diseases is highly accurate and reproducible, and more sensitive for variant detection, than exome sequencing. Genet. Med. 17, 253–261. 10.1038/gim.2014.172 25412400 PMC4572572

[B10] Dulla K. Slijkerman R. van Diepen H. C. Albert S. Dona M. Beumer W. (2021). Antisense oligonucleotide-based treatment of retinitis pigmentosa caused by USH2A exon 13 mutations. Mol. Ther. 29 (8), 2441–2455. 10.1016/j.ymthe.2021.04.024 33895329 PMC8353187

[B11] Fuster-García C. García-García G. González-Romero E. Jaijo T. Sequedo M. D. Ayuso C. (2017). USH2A Gene Editing Using the CRISPR System. Molecular Therapy. Nucleic Acids 8, 529–541. 10.1016/j.omtn.2017.08.003 28918053 PMC5573797

[B12] Gao F-J. Li J-K. Chen H. Hu F. Y. Zhang S. H. Qi Y. H. (2019). Genetic and clinical findings in a large cohort of Chinese patients with suspected retinitis pigmentosa. Ophthalmology 126, 1549–1556. 10.1016/j.ophtha.2019.04.038 31054281

[B13] Gao F. J. Wang D. D. Chen F. Sun H. X. Hu F. Y. Xu P. (2021). Prevalence and genetic-phenotypic characteristics of patients with USH2A mutations in a large cohort of Chinese patients with inherited retinal disease. Br. J. Ophthalmol. 105 (1), 87–92. 10.1136/bjophthalmol-2020-315878 32188678 PMC7788223

[B14] Hanany M. Rivolta C. Sharon D. (2020). Worldwide carrier frequency and genetic prevalence of autosomal recessive inherited retinal diseases. Proc. Natl. Acad. Sci. U. S. A. 117, 2710–2716. 10.1073/pnas.1913179117 31964843 PMC7007541

[B15] Horton J. S. Flanagan L. M. Jackson R. W. Priest N. K. Taylor T. B. (2021). A mutational hotspot that determines highly repeatable evolution can be built and broken by silent genetic changes. Nat. Commun. 12, 6092. 10.1038/s41467-021-26286-9 34667151 PMC8526746

[B16] Inaba A. Maeda A. Yoshida A. Kawai K. Hirami Y. Kurimoto Y. (2020). Truncating variants contribute to hearing loss and severe retinopathy in USH2A-associated retinitis pigmentosa in Japanese patients. Int. J. Mol. Sci. 21 (21), 7817. 10.3390/ijms21217817 PMC7659936 33105608

[B17] Jiang L. Liang X. Li Y. Wang J. Zaneveld J. E. Wang H. (2015). Comprehensive molecular diagnosis of 67 Chinese usher syndrome probands: High rate of ethnicity specific mutations in Chinese USH patients. Orphanet J. Rare Dis. 10, 110. 10.1186/s13023-015-0329-3 26338283 PMC4559966

[B18] Johnston J. J. Biesecker L. G. (2013). Databases of genomic variation and phenotypes: Existing resources and future needs. Hum. Mol. Genet. 22, R27–R31. 10.1093/hmg/ddt384 23962721 PMC3782073

[B19] Jouret G. Poirsier C. Spodenkiewicz M. Jaquin C. Gouy E. Arndt C. (2019). Genetics of usher syndrome: New insights from a meta-analysis. Otol. Neurotol. 40, 121–129. 10.1097/MAO.0000000000002054 30531642

[B20] Koyanagi Y. Akiyama M. Nishiguchi K. M. Momozawa Y. Kamatani Y. Takata S. (2019). Genetic characteristics of retinitis pigmentosa in 1204 Japanese patients. J. Med. Genet. 56 (10), 662–670. 10.1136/jmedgenet-2018-105691 31213501

[B21] Lenassi E. Vincent A. Li Z. Saihan Z. Coffey A. J. Steele-Stallard H. B. (2015). A detailed clinical and molecular survey of subjects with nonsyndromic USH2A retinopathy reveals an allelic hierarchy of disease-causing variants. Eur. J. Hum. Genet. 23 (10), 1318–1327. 10.1038/ejhg.2014.283 25649381 PMC4592079

[B22] Li W. Qu N. Li J. K. Li Y. X. Han D. M. Chen Y. X. (2021). Evaluation of the genetic variation spectrum related to corneal dystrophy in a large cohort. Front. Cell Dev. Biol. 9, 632946. 10.3389/fcell.2021.632946 33816482 PMC8012530

[B23] Liu X. Bulgakov O. V. Darrow K. N. Pawlyk B. Adamian M. Liberman M. C. (2007). Usherin is required for maintenance of retinal photoreceptors and normal development of cochlear hair cells. Proc. Natl. Acad. Sci. U. S. A. 104 (11), 4413–4418. 10.1073/pnas.0610950104 17360538 PMC1838616

[B24] McGee T. L. Seyedahmadi B. J. Sweeney M. O. Dryja T. P. Berson E. L. (2010). Novel mutations in the long isoform of the USH2A gene in patients with Usher syndrome type II or non-syndromic retinitis pigmentosa. J. Med. Genet. 47, 499–506. 20507924 10.1136/jmg.2009.075143 PMC3070405

[B25] Meng X. Liu X. Li Y. Guo T. Yang L. (2021). Correlation between genotype and phenotype in 69 Chinese patients with USH2A mutations: A comparative study of the patients with usher syndrome and nonsyndromic retinitis pigmentosa. Acta Ophthalmol. 99 (4), e447–e460. 10.1111/aos.14626 33124170

[B26] Millán J. M. Aller E. Jaijo T. Blanco-Kelly F. Gimenez-Pardo A. Ayuso C. (2011). An update on the genetics of Usher syndrome. J. Ophthalmol. 2011, 417217. 10.1155/2011/417217 21234346 PMC3017948

[B27] Nakanishi H. Ohtsubo M. Iwasaki S. Hotta Y. Usami S. I. Mizuta K. (2011). Novel USH2A mutations in Japanese usher syndrome type 2 patients: Marked differences in the mutation spectrum between the Japanese and other populations. J. Hum. Genet. 56 (7), 484–490. 10.1038/jhg.2011.45 21593743

[B28] Ng T. K. Tang W. Cao Y. Chen S. Zheng Y. Xiao X. (2019). Whole exome sequencing identifies novel USH2A mutations and confirms Usher syndrome 2 diagnosis in Chinese retinitis pigmentosa patients. Sci. Rep. 9 (1), 5628. 10.1038/s41598-019-42105-0 30948794 PMC6449333

[B29] Pennings R. J. Te Brinke H. Weston M. D. Claassen A. Orten D. J. Weekamp H. (2004). USH2A mutation analysis in 70 Dutch families with Usher syndrome type II. Hum. Mutat. 24 (2), 185. 10.1002/humu.9259 15241801

[B30] Perea-Romero I. Gordo G. Iancu I. F. Del Pozo-Valero M. Almoguera B. Blanco-Kelly F. (2021). Author Correction: Genetic landscape of 6089 inherited retinal dystrophies affected cases in Spain and their therapeutic and extended epidemiological implications. Sci. Rep. 11, 10340–10413. The ESRETNET Study Group. 10.1038/s41598-021-89275-4 33972629 PMC8110971

[B31] Pontikos N. Arno G. Jurkute N. Schiff E. Abbad B. R. Malka S. (2020). Genetic basis of inherited retinal disease in a molecularly characterised cohort of over 3000 families from the United Kingdom[J]. Ophthalmology 127, 1384–1394. 10.1016/j.ophtha.2020.04.008 32423767 PMC7520514

[B32] Puech B. Kostrubiec B. Hache J. C. François P. (1991). Epidemiology and prevalence of hereditary retinal dystrophies in the Northern France. J. Fr. Ophtalmol. 14, 153–164. 1918822

[B33] Qu L. H. Jin X. Long Y. L. Ren J. Y. Weng C. H. Xu H. W. (2020). Identification of 13 novel USH2A mutations in Chinese retinitis pigmentosa and Usher syndrome patients by targeted next-generation sequencing. Biosci. Rep. 40 (1), BSR20193536. 10.1042/BSR20193536 31904091 PMC6974426

[B34] Reurink J. Dockery A. Oziębło D. Farrar G. J. Ołdak M. Ten Brink J. B. (2021). Molecular inversion probe-based sequencing of USH2A exons and splice sites as a cost-effective screening tool in USH2 and arRP cases. Int. J. Mol. Sci. 22 (12), 6419. 10.3390/ijms22126419 34203967 PMC8232728

[B35] Richards S. Aziz N. Bale S. Bick D. Das S. Gastier-Foster J. (2015). Standards and guidelines for the interpretation of sequence variants: A joint consensus recommendation of the American College of medical genetics and genomics and the association for molecular pathology. Genet. Med. 17, 405–424. 10.1038/gim.2015.30 25741868 PMC4544753

[B36] Rosenberg T. Haim M. Hauch A.-M. Parving A. (2008). The prevalence of Usher syndrome and other retinal dystrophy-hearing impairment associations. Clin. Genet. 51, 314–321. 10.1111/j.1399-0004.1997.tb02480.x 9212179

[B37] Roux A. F. Faugère V. Vaché C. Baux D. Besnard T. Léonard S. (2011). Four-year follow-up of diagnostic service in USH1 patients. Invest. Ophthalmol. Vis. Sci. 52 (7), 4063–4071. 10.1167/iovs.10-6869 21436283

[B3] Samanta A. Stingl K. Kohl S. Ries J. Linnert J. Nagel-Wolfrum K. (2019). Ataluren for the Treatment of Usher Syndrome 2A Caused by Nonsense Mutations. Int. J. Mol. Sci. 20 (24), 6274. 10.3390/ijms20246274 PMC6940777 31842393

[B38] Sanjurjo-Soriano C. Erkilic N. Baux D. Mamaeva D. Hamel C. P. Meunier I. (2019). Genome editing in patient iPSCs corrects the most prevalent USH2A mutations and reveals intriguing mutant mRNA expression profiles. Mol. Ther. Methods Clin. Dev. 17, 156–173. 10.1016/j.omtm.2019.11.016 31909088 PMC6938853

[B39] Spandau U. H. Rohrschneider K. (2002). Prevalence and geographical distribution of Usher syndrome in Germany. Graefes Arch. Clin. Exp. Ophthalmol. 240, 495–498. 10.1007/s00417-002-0485-8 12107518

[B40] Stenson P. D. Ball E. V. Mort M. Phillips A. D. Shaw K. Cooper D. N. (2012). The Human Gene Mutation Database (HGMD) and its exploitation in the fields of personalized genomics and molecular evolution. Curr. Protoc. Bioinforma. 1, 13. Chapter 1Unit1.13. 10.1002/0471250953.bi0113s39 22948725

[B41] Stephen P. Daiger P. Lori S. Sullivan P. Sara J. Bowne P. RetNet: Retinal information network. Available at: https://sph.uth.edu/retnet/sum-dis.htm (Accessed December 31, 2021).

[B42] Stone E. M. Andorf J. L. Whitmore S. S. DeLuca A. P. Giacalone J. C. Streb L. M. (2017). Clinically focused molecular investigation of 1000 consecutive families with inherited retinal disease. Ophthalmology 124, 1314–1331. 10.1016/j.ophtha.2017.04.008 28559085 PMC5565704

[B43] Sun T. Xu K. Ren Y. Xie Y. Zhang X. Tian L. (2018). Comprehensive molecular screening in Chinese Usher syndrome patients. Invest. Ophthalmol. Vis. Sci. 59, 1229–1237. 10.1167/iovs.17-23312 29625443

[B44] Sun Y. Li W. Li J. K. Wang Z. S. Bai J. Y. Xu L. (2020). Genetic and clinical findings of panel-based targeted exome sequencing in a northeast Chinese cohort with retinitis pigmentosa. Mol. Genet. Genomic Med. 8 (4), e1184. 10.1002/mgg3.1184 32100970 PMC7196472

[B45] Toualbi L. Toms M. Moosajee M. (2020). USH2A-retinopathy: From genetics to therapeutics. Exp. Eye Res. 201, 108330. 10.1016/j.exer.2020.108330 33121974 PMC8417766

[B46] Van Wijk E. Pennings R. J. te Brinke H. Claassen A. Yntema H. G. Hoefsloot L. H. (2004). Identification of 51 novel exons of the Usher syndrome type 2A (USH2A) gene that encode multiple conserved functional domains and that are mutated in patients with Usher syndrome type II. Am. J. Hum. Genet. 74, 738–744. 10.1086/383096 15015129 PMC1181950

[B47] Wang K. Li M. Hakonarson H. (2010). Annovar: Functional annotation of genetic variants from high-throughput sequencing data. Nucleic Acids Res. 38 (16), e164. 10.1093/nar/gkq603 20601685 PMC2938201

[B48] Zhu T. Chen D. F. Wang L. Wu S. Wei X. Li H. (2021). USH2A variants in Chinese patients with Usher syndrome type II and non-syndromic retinitis pigmentosa. Br. J. Ophthalmol. 105 (5), 694–703. 10.1136/bjophthalmol-2019-315786 32675063

[B49] Zlotogora J. (1994). High frequencies of human genetic diseases: Founder effect with genetic drift or selection? Am. J. Med. Genet. 49 (1), 10–13. 10.1002/ajmg.1320490104 8172234

